# Awareness-Guided Self-Supervised Visual Odometry via Fusion of Pseudo-Depth and Residual-Flow Cues

**DOI:** 10.3390/s26185954

**Published:** 2026-09-20

**Authors:** Shi Zhou, Zijun Yang, Zhen Li, Xianchang Li, Yuchen Sun, Lifeng Zhang

**Affiliations:** 1 Huzhou Key Laboratory of Green Energy Materials and Battery Cascade Utilization, School of Intelligent Manufacturing, Huzhou College, Wuxing, Huzhou 313000, China; 2 College of Digital Technology and Engineering, Ningbo University of Finance and Economics, Haishu, Ningbo 315000, China; 3 Department of Electrical and Electronic Engineering, Kyushu Institute of Technology, Tobata, Kitakyushu 8040015, Fukuoka, Japan

**Keywords:** visual odometry, ego-motion estimation, self-supervised learning, scene awareness, multimodal fusion

## Abstract

**Highlights:**

**What are the main findings?**

- Pseudo-depth and residual flow provide complementary geometric and motion cues for identifying unreliable cross-frame observations caused by non-observable regions and independently moving objects.
- Incorporating awareness of reliable cross-frame observations into self-supervised pose learning at both the input and loss levels improves ego-motion estimation and reduces trajectory errors.

**What are the implications of the main findings?**

- Awareness of the reliability of cross-frame observations provides a more effective basis for self-supervised pose learning than treating all image regions as equally reliable.
- Combining complementary geometric and motion information offers a practical way to improve the accuracy of self-supervised visual odometry.

**Abstract:**

Self-supervised monocular visual odometry (VO) commonly relies on photometric consistency and rigid-scene assumptions to learn camera motion, making pose estimation vulnerable to unreliable cross-frame observations caused by limited geometric observability and independently moving objects. To address this problem, we propose an awareness-guided self-supervised visual odometry framework that emphasizes reliable cross-frame observations for pose learning. Pseudo-depth is exploited to characterize geometric observability and identify regions that are unreliable for cross-frame reconstruction, while residual flow is employed to capture motion inconsistency introduced by independently moving objects. The resulting observable static regions are incorporated into pose estimation at both the input and loss levels, enabling unreliable observations to be suppressed before feature extraction and during optimization. Qualitative experiments on KITTI and Cityscapes verify the effectiveness of pseudo-depth and residual flow in extracting geometric observability and dynamic-motion information across different driving scenes. Quantitative experiments on the KITTI Odometry dataset show that the proposed method achieves average relative translation and rotation errors of 6.04% and 2.42°/100 m, together with an ATE of 0.88 m and an ARE of 0.20°, demonstrating improved ego-motion and trajectory estimation.

## 1. Introduction

Reliable ego-motion estimation is a prerequisite for autonomous navigation, trajectory recovery, environmental perception, and geometric scene reconstruction [1,2,3,4,5,6]. Visual odometry (VO) estimates the motion of a camera from sequential visual observations and therefore provides an important motion source when external positioning signals are unavailable or unreliable. Self-supervised VO has received increasing attention because it learns depth and relative pose directly from unlabeled image sequences, substantially reducing the need for ground-truth trajectories [7,8]. Most methods obtain supervision through differentiable view synthesis: depth and pose predictions are used to warp one frame into another, and the resulting photometric or geometric discrepancy drives learning.

This learning principle nevertheless rests on two coupled assumptions. First, the observed scene should be approximately rigid, so that image motion can be explained by camera ego-motion and scene depth. Second, the pixels used by the loss should remain geometrically observable across the considered frames. Complex traffic scenes violate both assumptions. Vehicles, pedestrians, and cyclists may move independently of the camera, causing object motion to be absorbed incorrectly into the estimated ego-motion [9,10]. At the same time, some nominally static pixels still fail to provide reliable constraints. Pixels projected outside the adjacent image have no valid photometric counterpart, and near-camera ground pixels can exhibit large image displacement and high sensitivity to small depth or pose perturbations. Consequently, static appearance alone is not a sufficient criterion for valid pose supervision.

Existing methods mitigate unreliable supervision through robust reconstruction losses, minimum reprojection, auto-masking, semantic filtering, uncertainty weighting, or motion-consistency constraints [11,12,13,14,15,16,17,18,19,20]. These strategies have improved self-supervised depth and pose learning, but their treatment of reliability is often partial. Dynamic-object methods primarily target violations of scene rigidity, whereas validity masks commonly address image boundaries or photometric outliers. The broader distinction between observable static regions and unreliable cross-frame observations is therefore rarely made explicit. This distinction matters because dynamic motion and limited observability arise from different mechanisms and require different cues.

We address this issue by introducing an awareness-guided self-supervised monocular VO framework that focuses pose learning on reliable cross-frame observations. Given adjacent monocular frames, the framework estimates pseudo-depth, residual-flow, and coarse camera motion to characterize geometric observability and motion consistency. The pseudo-depth information is used to identify non-observable regions with unreliable cross-frame geometric constraints, while the residual flow is exploited to detect independently moving regions that violate the rigid-scene assumption. By excluding these unreliable observations, the remaining observable static regions provide more reliable cross-frame constraints for camera pose estimation. This awareness is incorporated into pose learning at both the input and loss levels, allowing unreliable observations to be suppressed before feature extraction and during self-supervised optimization.

The main contributions are summarized as follows:

- We propose an awareness-guided self-supervised monocular VO framework that explicitly distinguishes reliable cross-frame observations from unreliable ones, allowing camera pose estimation to be guided by observable static scene regions.
- We exploit pseudo-depth and residual flow as complementary geometric and motion cues to identify non-observable and dynamic regions, respectively, and incorporate the resulting awareness at both the input and loss levels.
- Extensive experiments validate the effectiveness of the proposed framework. Quantitative evaluations on the KITTI Odometry dataset demonstrate improved ego-motion estimation, while qualitative results on KITTI and Cityscapes verify the effectiveness of pseudo-depth-based geometric information and residual-flow-based motion information in identifying unreliable cross-frame observations.

## 2. Related Work

### 2.1. Visual Odometry and Self-Supervised Learning

Classical VO estimates camera motion by establishing cross-frame correspondences and solving a geometric optimization problem. Feature-based systems, including ORB-SLAM and its extensions [21,22,23], combine sparse keypoint matching with pose optimization and bundle adjustment. Direct methods, such as LSD-SLAM [24] and Direct Sparse Odometry [25], optimize photometric residuals over selected pixels without explicit descriptors. Beyond autonomous driving, visual localization and SLAM have also been applied to complex robotic operating environments. For example, Chen et al. [26] integrated SLAM-based real-time localization and multi-map fusion into a visual servo framework for continuous fruit-harvesting operations in large-scale unstructured orchards. Such applications further highlight the importance of reliable camera motion estimation under complex scene structures and continuously changing observations. However, classical geometric approaches generally rely on sufficiently stable visual correspondences, and their performance may degrade under weak texture, severe appearance variation, occlusion, or independently moving objects.

Learning-based VO shifts part of the estimation problem from hand-designed correspondence processing to data-driven representation learning. Zhou et al. [27] established the widely adopted self-supervised view-synthesis paradigm for joint monocular depth and pose estimation. Subsequent methods improved the quality of supervision through minimum reprojection and auto-masking [14], joint depth-flow-motion modeling [15,16], uncertainty-aware direct optimization [17], cross-view geometric reasoning [18], iterative depth-pose refinement [19], and strengthened pose constraints [20]. Collectively, these studies show that depth and pose are tightly coupled: biased depth degrades pose-based warping, while inaccurate pose produces misleading depth supervision.

Despite these advances, photometric consistency remains an indirect supervisory signal. A low residual may arise from correct geometry, but also from low texture or accidental appearance agreement; a high residual may result from object motion, occlusion, illumination change, or an invalid projection. Robust pose learning therefore requires reasoning about whether a pixel can support the assumed geometric model, rather than treating the residual alone as a complete measure of correspondence quality.

### 2.2. Unreliable Observations in Dynamic Traffic Scenes

Independently moving objects violate the rigid-scene assumption underlying both geometric reprojection and photometric pose learning [28,29,30]. If their image motion is interpreted as camera motion, the resulting supervision can bias pose estimates and amplify trajectory drift. Semantic approaches identify potentially dynamic categories and suppress them during tracking or optimization [11,31,32]. Their effectiveness, however, depends on additional networks or labeled data, and semantic category does not necessarily determine the actual motion state; for example, a parked vehicle and a moving vehicle share a class but impose different geometric consequences [33,34].

Motion-based approaches instead detect disagreement between observed motion and the motion predicted by rigid geometry. Optical flow, epipolar inconsistency, photometric residuals, and forward-backward consistency have all been used to identify independently moving pixels [15,16,35,36,37,38,39,40,41]. These cues are closer to the physical source of the violation, but they can be noisy in textureless areas, under illumination change, or around occlusion boundaries.

Dynamic-region suppression alone is insufficient because a static point may still be unobservable in an adjacent frame. In projective view synthesis, a pixel has valid support only if its transformed coordinate lies inside the source image and the corresponding surface remains visible. Moreover, the conditioning of this constraint varies spatially. Near-camera ground points often undergo larger image displacement for a given pose perturbation than distant background points; depth and pose errors therefore produce disproportionately large reprojection errors in these regions. This paper treats these out-of-view and pose-sensitive pixels as non-observable for the purpose of pose supervision, thereby extending reliability analysis beyond semantic or motion state.

### 2.3. Geometric and Motion Cues for Reliable Pose Learning

Depth and optical flow provide complementary information for reasoning about cross-frame observations. Depth establishes the geometric relationship between image pixels and the 3D scene, allowing the projected support of a pixel under camera motion to be evaluated. Optical flow, in contrast, directly describes image-space motion and can reveal motion components that cannot be explained by the rigid-camera model. Previous studies have exploited these cues for joint depth, flow, and ego-motion estimation [15,16], geometric consistency learning [7], feature reconstruction [42], and correspondence selection [43,44]. These studies demonstrate the benefit of incorporating complementary geometric and motion information into self-supervised scene understanding.

Geonet [15] exploits residual motion by decomposing optical flow into rigid and residual components, where the residual flow is used to model non-rigid-scene motion and improve full-flow reconstruction. The residual flow in our method is not introduced to refine the optical-flow field. Instead, it is used as a motion-consistency cue to identify observations that deviate from the rigid-scene assumption and are therefore less reliable for pose estimation.

Beyond motion inconsistency, reliable pose learning also requires consideration of geometric observability. In our framework, the depth cue identifies cross-frame observations that are invalid or sensitive to projection errors, whereas the residual-flow cue identifies observations whose motion is inconsistent with rigid camera motion. Their combination defines the observable static support used for pose learning.

## 3. Materials and Methods

### 3.1. Framework Overview

**Figure 1:**
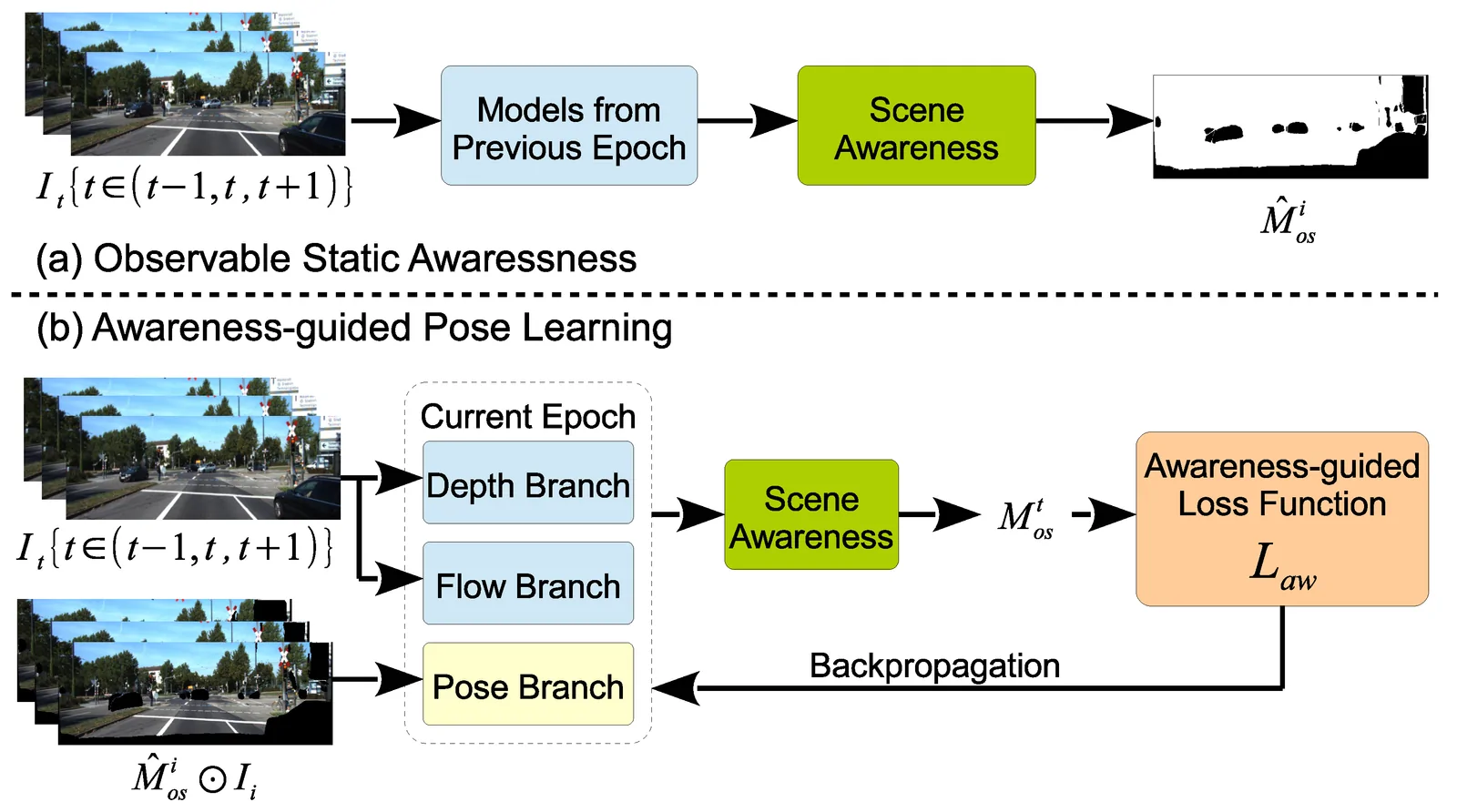
Overview of the proposed awareness-guided self-supervised visual odometry framework.

The proposed framework improves self-supervised monocular VO by constraining pose learning to image regions that are both static and geometrically observable. As illustrated in Figure 1, the framework consists of two stages: (a) observable static awareness and (b) awareness-guided pose learning.

In the observable static awareness stage, the original monocular image sequence $\left\{I_{i},i\in \left(t-1,t,t+1\right)\right\}$ is fed into the model obtained from the previous epoch. Based on the scene-awareness module described in Section 3.3, an initial observable static mask $M_{os}^{i}$ is constructed to identify regions that provide reliable geometric cues for camera motion estimation.

In the awareness-guided pose learning stage, the pseudo-depth and residual-flow estimation branches still take the original monocular image sequence as input, whereas the pose estimation branch receives the masked sequence $M_{os}^{i}⊙I_{i}$, where ⊙ denotes element-wise multiplication. This input-level guidance restricts the pose estimation branch to extracting motion-related features primarily from observable static regions. Meanwhile, the observable static mask $M_{os}^{t}$ corresponding to current epoch models is constructed according to the same scene-awareness module. During mask construction, the outputs of the three networks are detached from the computational graph, ensuring that the mask generation process introduces neither additional gradient paths nor circular supervision. The resulting $M_{os}^{t}$ is subsequently incorporated into the awareness-guided loss to provide loss-level guidance, thereby suppressing unreliable supervision from dynamic objects, out-of-view areas, and pose-sensitive regions during view reconstruction. Through this dual-guidance strategy, the proposed framework directs pose learning toward geometrically reliable observable static regions at both the network-input and loss-supervision levels.

### 3.2. Self-Supervised Learning of Geometric and Motion Cues

**Figure 2:**
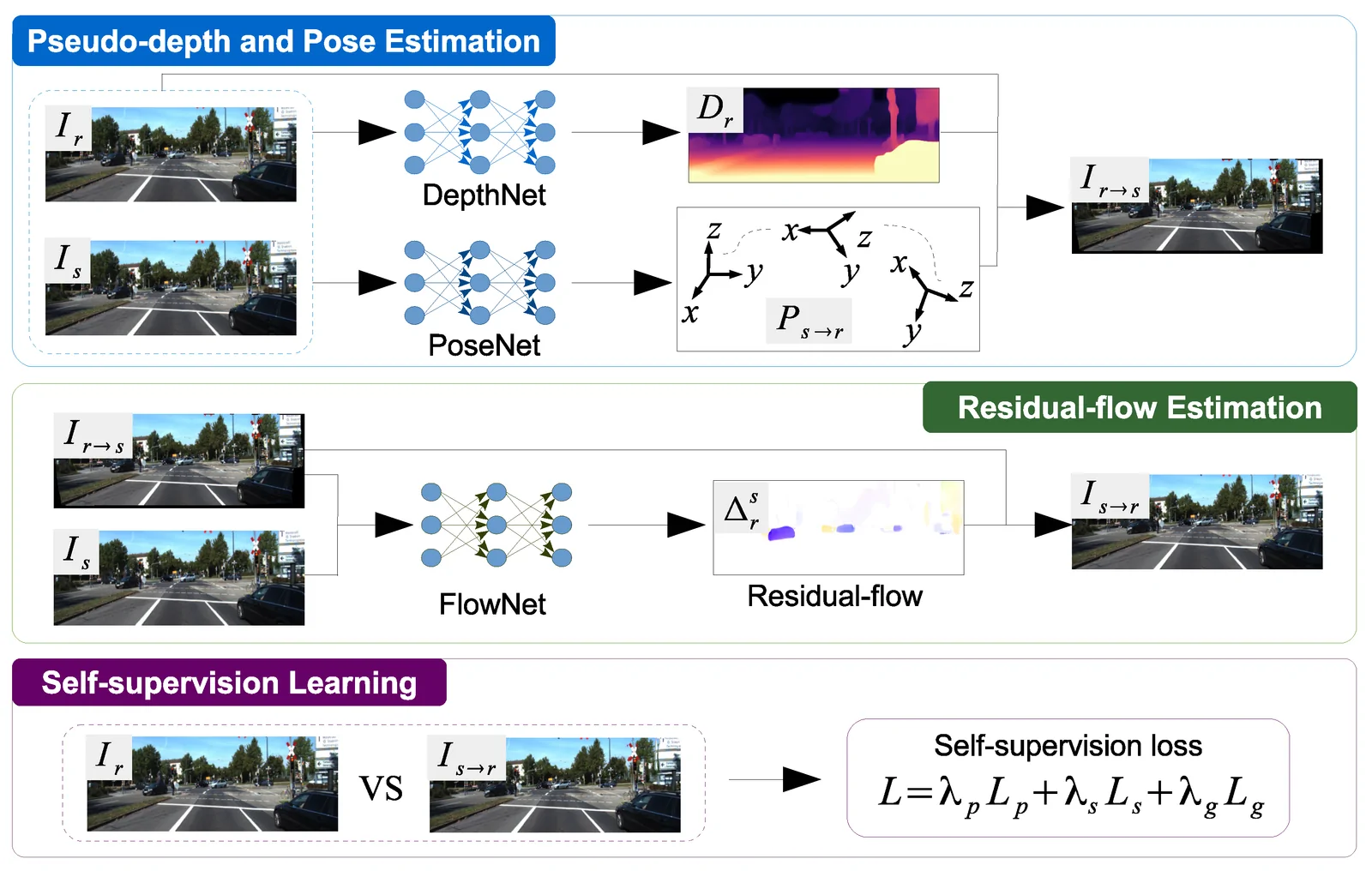
Self-supervised learning module for pseudo-depth, residual flow, and camera pose estimation. The module learns geometric and motion cues from monocular image sequences through view reconstruction without external annotations. The input to PoseNet differs between the two training stages described in Section 4.2. In the first stage, PoseNet takes the original image sequence as input, whereas in the second stage, it takes the masked sequence $M_{os}^{i}⊙I_{t}$, t∈{r,s} as input to focus pose estimation on observable static regions.

Monocular image sequences provide rich appearance information, but 3D scene structure and inter-frame motion cannot be directly obtained from a single image. To fuse geometric and motion cues from depth map and optical flow, we construct a self-supervised learning module that estimates pseudo-depth, residual flow, and pose from monocular sequences, as shown in Figure 2. The module contains three estimation branches for pseudo-depth, camera pose, and residual flow, referred to as DepthNet, PoseNet, and FlowNet, respectively. The network architectures of DepthNet and PoseNet follow the corresponding implementations of Monodepth2 [14], while FlowNet is based on RAFT [45]. During training, three consecutive frames are used for bidirectional loss computation and observable static awareness construction, while each network processes the corresponding adjacent frame pairs independently.

Let $I_{r}$ denote a reference frame and $I_{s}$ a temporally adjacent source frame. DepthNet predicts a dense pseudo-depth map $D_{r}$ for $I_{r}$, and PoseNet estimates the relative transformation $P_{s\to r}$. Given the camera intrinsic matrix *K*, the source view is reconstructed through differentiable projective warping:

(1) $$I_{r\to s}=\Phi \langle K,D_{r},I_{r},P_{s\to r}\rangle ,$$

where $\Phi \langle \cdot \rangle$ denotes back-projection of reference pixels into 3D, transformation by $P_{s\to r}$, projection into the source image, and differentiable sampling. Under rigid motion, correct depth, and valid visibility, $I_{r\to s}$ should be photometrically consistent with $I_{s}$.

The residual between the rigidly reconstructed and captured views contains information about model violations, including independent object motion. FlowNet therefore receives these two views and predicts a residual-flow field expressed in the reference frame coordinate system:

(2) $$\Delta_{r}^{s}=F\left(I_{r\to s},I_{s}\right).$$

This residual flow should be distinguished from raw optical flow between $I_{r}$ and $I_{s}$. Raw flow contains both rigid camera-induced displacement and non-rigid motion, and residual flow is intended to retain only the portion not explained by the depth–pose reconstruction. The distinction is central to the dynamic region detector.

Using the estimated residual flow $\Delta_{r}^{s}$ of reference image, an inverse warping operation $\Psi \langle \cdot \rangle$ is further performed to reconstruct the reference image from the source frame:

(3) $$I_{s\to r}=\Psi \langle I_{s},D_{r},\Delta_{r}^{s}\rangle .$$

This formulation provides an additional self-supervised signal that couples pseudo-depth, pose, and residual-flow learning. The loss function consists of photometric consistency loss $L_{p}$ [14], depth smoothness loss $L_{s}$ [46], and geometric consistency loss $L_{g}$ [47]:

(4) $$L=\lambda_{p}L_{p}\left(I_{s\to r},I_{r}\right)+\lambda_{s}L_{s}\left(D_{r},I_{r}\right)+\lambda_{g}L_{g}\left(\Delta_{r}^{s}\right),$$

where $\lambda_{p}$, $\lambda_{s}$, and $\lambda_{g}$ are loss weights, with a default set of 1.0, 0.002, and 0.02, respectively, following the setting in [48]. $L_{p}$ jointly constrains pseudo-depth, pose, and residual flow through view reconstruction, $L_{s}$ regularizes pseudo-depth while preserving image discontinuities, and $L_{g}$ penalizes unnecessary residual motion by encouraging $\Delta_{r}^{s}$ toward zero.

### 3.3. Observable Static Awareness

A pixel is considered reliable only when it satisfies two complementary conditions: (a) it is geometrically observable; (b) its observed motion is compatible with the rigid-scene assumption. Accordingly, pseudo-depth and camera motion are used to evaluate geometric observability, while residual flow is used to assess rigid-motion compatibility. The resulting observable static mask is considered the result of scene awareness.

#### 3.3.1. Non-Observable Region Detection

**Figure 3:**
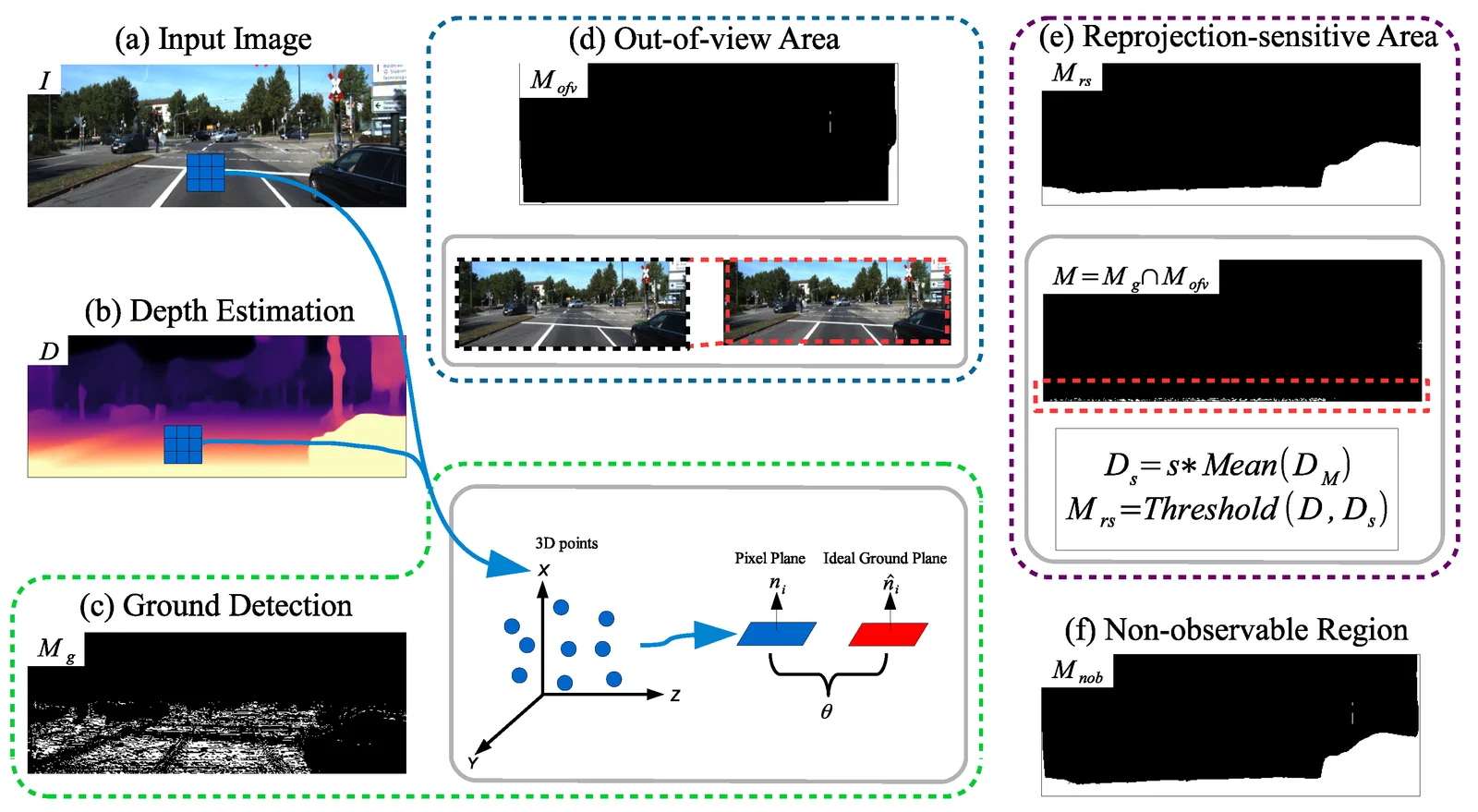
Depth-based non-observable region detection. Ground regions are first detected by comparing local surface normals with the ideal ground-plane normal. Out-of-view areas are identified by depth-based reconstruction across adjacent frames. Reprojection-sensitive areas are then determined from near-camera ground regions using an adaptive depth threshold. The final non-observable mask is obtained by combining the out-of-view and reprojection-sensitive masks.

The vehicle-borne camera is mounted at a fixed height, and its imaging geometry provides a useful prior for analyzing the relationship between the camera and the road surface. As shown in Figure 3, each reference pixel $p_{i}$ with pseudo-depth $D_{i}$, the corresponding 3D point is obtained by back-projection:

(5) $$P_{i}=\mathrm{Tr}\left(p_{i},K,D_{i}\right),$$

where Tr(·) denotes the depth-based back-projection from image coordinates to 3D space.

Based on this geometric prior, ground regions are detected by analyzing the spatial distribution of the reconstructed 3D points and measuring the consistency between the local surface normal and the ideal ground-plane normal, as shown in Figure 3c. For each pixel $p_{i}$, its eight neighboring pixels are divided into four overlapping subsets, each forming a local surface plane with the center pixel in 3D space. A unit normal is computed for each local surface plane, and the surface normal $n_{i}$ of pixel $p_{i}$ is obtained by averaging the four unit normals. Let ${\hat{n}}_{i}$ denote the ideal ground-plane normal determined by the camera mounting configuration. The angular deviation between $n_{i}$ and ${\hat{n}}_{i}$ is

(6) $$\theta =\left|\arccos(n_{i}\cdot {\hat{n}}_{i})\right|.$$

Pixels whose angular deviation is smaller than a predefined threshold are classified as ground pixels. In this study, the threshold is set to $5^{\circ }$ following [49].

As the vehicle moves forward, reconstructing the previous frame from the current frame may cause pixels near the image boundaries to lose valid correspondences when their projected locations fall outside the available image domain, as shown in Figure 3d. This type of failure is different from reconstruction errors within the image interior, where occlusion, illumination variation, or depth discontinuities may still affect photometric consistency. In the interior region, most pixels retain sufficient neighboring samples to support grid-sampling. However, boundary pixels often suffer from incomplete local support because part of their neighborhood lies outside the image. This lack of contextual information can lead to noticeable reconstruction distortion or missing content. Therefore, these boundary regions provide unreliable geometric constraints for pose estimation.

To identify such regions, each pixel p(u,v) in the current frame is projected into the previous frame using the estimated pseudo-depth $D_{t}\left(p\right)$, the camera intrinsic matrix *K*, and the relative coarse pose $P_{t\to t+1}$:

(7) $$p^{\prime }\left(u^{\prime },v^{\prime }\right)=\Phi \langle K,D_{t}\left(p\right),p\left(u,v\right),P_{t\to t+1}\rangle .$$

If the projected coordinate $p^{\prime }\left(u^{\prime },v^{\prime }\right)$ lies outside the image domain Ω, the corresponding pixel is assigned to the out-of-view mask $M_{ofv}$:

(8) $$M_{ofv}=1\left[p^{\prime }\notin \Omega \right],$$

where 1[·] is indicator function. The mask value of 1 indicates that the projected pixel falls outside the image domain and is therefore classified as an out-of-view pixel. Such a pixel has no valid sampling support in the adjacent frame and should not contribute to photometric pose supervision.

In addition, as shown in Equation (7), the inter-frame reprojection jointly depends on the estimated pseudo-depth and camera pose. Pixels close to the camera are generally more sensitive to camera motion and can therefore provide strong motion constraints when the geometry and pose are accurately estimated. However, during self-supervised learning, both the estimated pose and pseudo-depth inevitably contain errors. For near-camera regions, even small estimation errors may result in large reprojection deviations, leading to noticeable misalignment during view synthesis and unstable photometric supervision. To reduce the influence of such error amplification on pose learning, these reprojection-sensitive regions are excluded from pose supervision.

To capture this geometric tendency, we derive an adaptive depth threshold $D_{s}$ from the detected ground pixels associated with the out-of-view region:

(9) $$M=M_{g}\cap M_{ofv},$$

(10) $$D_{s}=\left\{\begin{array}{cc} s\mathrm{Mean}(D(p)|p\in M) & M\neq \emptyset \\ 0 & M=\emptyset \end{array}\right.,$$

(11) $$M_{rs}=1\left[D\left(p\right)<D_{s}\right].$$

where *s* is a scaling coefficient. Pixels with depth values smaller than $D_{s}$ are classified as reprojection-sensitive. Thus, the final non-observable mask $M_{nob}$ is obtained by combining the out-of-view mask and the reprojection-sensitive mask:

(12) $$M_{nob}=M_{ofv}\cup M_{rs}.$$

This mask identifies regions that are difficult to observe reliably across consecutive frames and should therefore be suppressed during awareness-guided pose estimation.

#### 3.3.2. Dynamic Regions Detection

**Figure 4:**
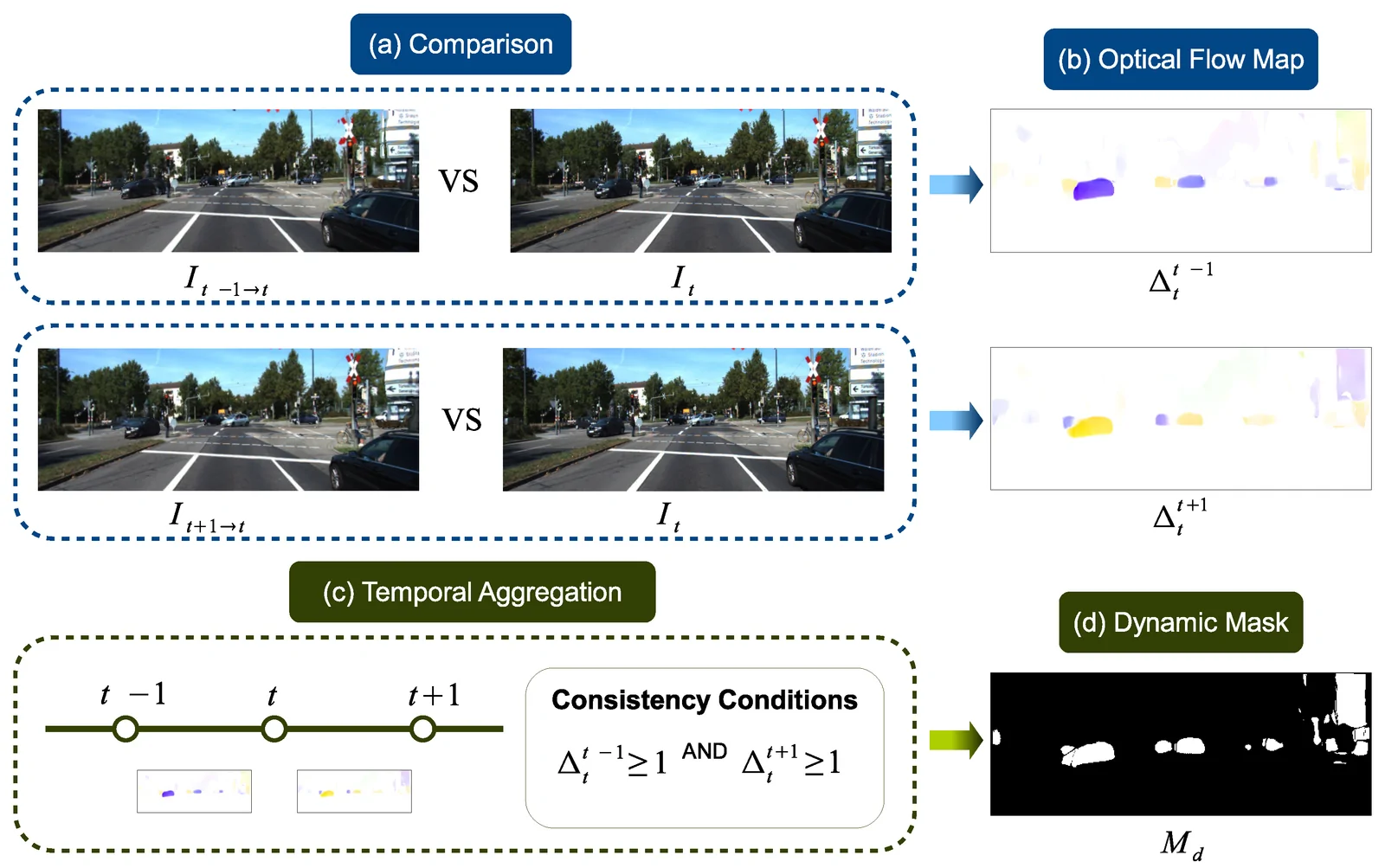
Residual-flow-based dynamic region detection. Residual flow is estimated by comparing reconstructed target frames with the captured target frame. Residual motion responses from both temporal directions are aggregated under consistency constraints. Pixels with residual motion in both directions are identified as dynamic regions and suppressed during awareness-guided pose estimation.

Dynamic objects violate the rigid-scene assumption of self-supervised VO, introducing motion that cannot be explained by camera ego-motion and thus misleading pose supervision. To reduce this influence, we use residual flow as a motion cue to identify dynamic regions, as shown in Figure 4.

Given the target frame $I_{t}$ and its adjacent frames $I_{t-1}$ and $I_{t+1}$, the adjacent frames are first warped into the target view according to the pseudo-depth and pose, producing $I_{t-1\to t}$ and $I_{t+1\to t}$. Based on these rigidly reconstructed images, FlowNet F(·) predicts the residual motion with respect to $I_{t}$ in the target image coordinate system:

(13) $$\Delta_{t}^{t-1}=F\left(I_{t-1\to t},I_{t}\right),$$

(14) $$\Delta_{t}^{t+1}=F\left(I_{t+1\to t},I_{t}\right),$$

For reliable static regions, most inter-frame motion can be explained by camera ego-motion, and the residual-flow response should remain weak. In contrast, independently moving objects usually produce residual motion that persists across adjacent temporal directions. Based on this observation, the dynamic decision is therefore based on the Euclidean magnitude of the residual flow in the two temporal directions:

(15) $$M_{d}\left(p\right)=1\left[∥\Delta_{t}^{t-1}\left(p\right)∥\geq 1\;\mathrm{and}\;∥\Delta_{t}^{t+1}\left(p\right)∥\geq 1\right].$$

This temporal consistency strategy suppresses transient noise and weak motion responses while preserving regions corresponding to independently moving objects. The resulting dynamic mask $M_{d}$ identifies moving objects, such as vehicles and pedestrians, that should be excluded from pose optimization. By suppressing these dynamic regions, the pose estimation module can rely more on static scene structures with stable cross-frame correspondences.

#### 3.3.3. Observable Static Region Detection

The observable static region is obtained by excluding both non-observable regions and dynamic regions from the image. Specifically, the non-observable mask $M_{nob}$ identifies out-of-view and reprojection-sensitive regions, while the dynamic mask $M_{d}$ identifies independently moving objects. The remaining pixels are regarded as observable static regions because they are more likely to maintain reliable cross-frame correspondences and provide stable geometric constraints for camera motion estimation.

The observable static mask can be expressed as the complement of the union of $M_{nob}$ and $M_{d}$:

(16) $$M_{os}=1-\left(M_{nob}\cup M_{d}\right).$$

These observable static regions are subsequently used to guide pose estimation, reducing the influence of unreliable observations and allowing the VO model to learn ego-motion mainly from reliable static scene structures.

### 3.4. Awareness-Guided Pose Estimation

After constructing the scene awareness representation, we incorporate it into pose estimation from both the input and optimization perspectives. The objective is to guide the PoseNet to focus on reliable observable static regions, where cross-frame correspondences are more stable and the motion is more consistent with vehicle ego-motion. In contrast, dynamic regions and non-observable regions are suppressed to reduce misleading supervision during self-supervised VO learning.

Under the rigid-scene assumption, camera motion is a frame-level variable shared by all observable static pixels, whereas pseudo-depth and residual flow remain pixel-wise quantities. Based on this distinction, different input strategies are adopted for PoseNet, DepthNet, and FlowNet. For PoseNet, the observable static masks are imposed at the input level to restrict motion estimation to geometrically reliable and static regions. In contrast, DepthNet and FlowNet operate on the complete images, preserving the original spatial appearance and local continuity required for dense pixel-wise prediction. This design avoids introducing artificial discontinuities at mask boundaries, which could otherwise distort depth and residual-flow estimation.

Given a reference frame $I_{r}$ and a source frame $I_{s}$, the observable static masks ${\hat{M}}_{os}^{r}$ and ${\hat{M}}_{os}^{s}$, generated by the model from the previous epoch, are applied to the corresponding images through element-wise multiplication before being fed into PoseNet:

(17) $$P_{r\to s}=\Gamma \left({\hat{M}}_{os}^{r}⊙I_{r},\;{\hat{M}}_{os}^{s}⊙I_{s}\right),$$

where ⊙ denotes element-wise multiplication and Γ(·) represents PoseNet. Since ${\hat{M}}_{os}^{r}$ and ${\hat{M}}_{os}^{s}$ are generated by the model from the previous epoch, they are independent of the computational graph of the current epoch. This input-level guidance suppresses dynamic and poorly observable regions before feature extraction, encouraging PoseNet to estimate ego-motion mainly from reliable static scene structures.

With the estimated relative pose $P_{r\to s}$, pseudo-depth $D_{r}$, and camera intrinsic matrix *K*, the source frame is warped into the reference view to obtain the reconstructed image $I_{s\to r}$. During the current epoch, a new observable static mask $M_{os}^{r}$ is generated from the current depth, pose, and residual-flow predictions. Before being used for loss computation, $M_{os}^{r}$ is explicitly detached from the computational graph and treated as a fixed binary mask. Therefore, no gradient is propagated through the mask-generation process. The awareness-guided loss is then defined as

(18) $$L_{aw}=\lambda_{p}\frac{\sum_{p}M_{os}^{r}\left(p\right)L_{p}\left(p\right)}{\sum_{p}M_{os}^{r}\left(p\right)+\varepsilon }+\lambda_{s}L_{s}+\lambda_{g}\frac{\sum_{p}M_{os}^{r}\left(p\right)L_{g}\left(p\right)}{\sum_{p}M_{os}^{r}\left(p\right)+\varepsilon },$$

where $M_{os}^{r}\left(p\right)\in \left\{0,1\right\}$ indicates whether pixel *p* belongs to the observable static region, and ε is a small positive constant introduced to avoid division by zero.

Through this dual-awareness-guided design, the observable static regions constrain pose learning at both the input and loss levels. The mask generated from the previous epoch guides PoseNet to extract motion cues from reliable static structures, while the detached mask generated in the current epoch prevents dynamic and poorly observable regions from contributing misleading photometric and geometric supervision. In this way, the proposed method reduces the influence of unreliable scene regions on pose learning and improves the accuracy of monocular VO in complex traffic scenes.

## 4. Results

### 4.1. Datasets and Evaluation Metrics

Experiments were conducted on the KITTI Odometry dataset [50], which provides driving sequences with ground-truth camera trajectories and is widely used for evaluating visual odometry. Following the commonly adopted self-supervised monocular VO protocol, Sequences 00–08 were used for training, while Sequences 09 and 10 were reserved for trajectory evaluation. To further assess the generalization capability of the proposed method, we additionally performed qualitative experiments on the Cityscapes dataset [51].

The estimated trajectories were evaluated using four metrics, including the relative translation error $t_{err}$, relative rotation error $r_{err}$, absolute translation error (ATE), and absolute rotation error (ARE). The first two metrics quantify the accumulated translational and rotational drift over local trajectory segments, whereas ATE and ARE measure the global discrepancy between the estimated and ground-truth trajectories.

Let $Q_{i}$ and $P_{i}$ denote the ground truth and estimated poses at frame *i*, respectively. Following [50], the relative trajectory errors are evaluated over eight segment lengths,

(19) L=100,200,300,400,500,600,700,800m,

for each trajectory segment of length $l_{s}\in L$, the poses within the segment are sampled at intervals of *k* frames, where *k* is set to 10 by default. The relative translation error is then calculated as:

(20) $$t_{err}=\frac{100}{\left|V\right|}\sum_{(i,s)\in V}\frac{{∥\mathrm{trans}\left[{\left(Q_{i}^{-1}Q_{i+k}\right)}^{-1}\left(P_{i}^{-1}P_{i+k}\right)\right]∥}_{2}}{l_{s}},$$

where V denotes the set of all valid evaluated trajectory segments and |V| is the total number of such segments. The operator trans(·) extracts the translation component of the relative pose error. Therefore, $t_{err}$ represents the average translational drift normalized by the traveled distance and is reported as a percentage (%).

Similarly, the relative rotation error is computed as:

(21) $$r_{err}=\frac{100}{\left|V\right|}\sum_{(i,s)\in V}\frac{\mathrm{angle}\left\{\mathrm{rot}\left[{\left(Q_{i}^{-1}Q_{i+k}\right)}^{-1}\left(P_{i}^{-1}P_{i+k}\right)\right]\right\}}{l_{s}},$$

where rot(·) extracts the rotation matrix from the relative pose error and angle(·) returns the corresponding rotation angle in degrees. Accordingly, $r_{err}$ represents the average rotational drift normalized by the traveled distance and is reported in degrees per 100 m (°/100 m).

To further evaluate the global trajectory accuracy, the estimated trajectory is scale-aligned with the ground-truth trajectory following [19]. For each evaluated trajectory, a single scale factor is obtained by least-squares fitting of the estimated translations to the ground-truth translations and is uniformly applied to the entire estimated trajectory. Let ${\tilde{P}}_{i}$ denote the aligned estimated pose at frame *i*. The absolute translation error (ATE) is calculated as:

(22) $$\mathrm{ATE}=\frac{1}{N}\sum_{i=1}^{N}{∥\mathrm{trans}\left(Q_{i}^{-1}{\tilde{P}}_{i}\right)∥}_{2},$$

where *N* is the total number of evaluated poses. ATE measures the global translational discrepancy between the aligned estimated trajectory and the ground-truth trajectory and is reported in meters (m).

The absolute rotation error (ARE) is computed from the rotation component of the absolute pose error as:

(23) $$\mathrm{ARE}=\frac{1}{\left|N\right|}\sum_{i=1}^{N}\mathrm{angle}\left\{\mathrm{rot}\left(Q_{i}^{-1}{\tilde{P}}_{i}\right)\right\},$$

ARE measures the global rotational discrepancy between the aligned estimated trajectory and the ground-truth trajectory and is reported in degrees (°).

### 4.2. Training Details

The framework was trained in two stages. In the first stage, DepthNet, PoseNet, and FlowNet were jointly trained for 15 epochs using the complete image observations with an initial learning rate of $1\times 10^{-4}$. This stage is introduced because the observable static masks are constructed from the pseudo-depth, residual flow, and camera pose predicted by the network. During the early stage of training, these predictions are not sufficiently reliable to provide stable region selection. Applying the masks from the beginning therefore suppresses informative pixels according to inaccurate geometric or motion estimates and propagates such errors back to pose learning.

After 15 epochs, the predicted depth, residual flow, and camera motion become sufficiently stable to provide more reliable observable static regions. Awareness-guided pose learning was then activated for another 10 epochs, with the learning rate reduced to $1\times 10^{-5}$ for fine-tuning. During this stage, the observable static mask is applied at both the input and loss levels. At the input level, unreliable regions are suppressed before pose inference, encouraging PoseNet to estimate camera motion primarily from geometrically observable and rigid-consistent regions. At the loss level, the self-supervised objective is restricted to the same reliable regions, thereby reducing the influence of dynamic objects and geometrically unstable observations.

**Figure 5:**
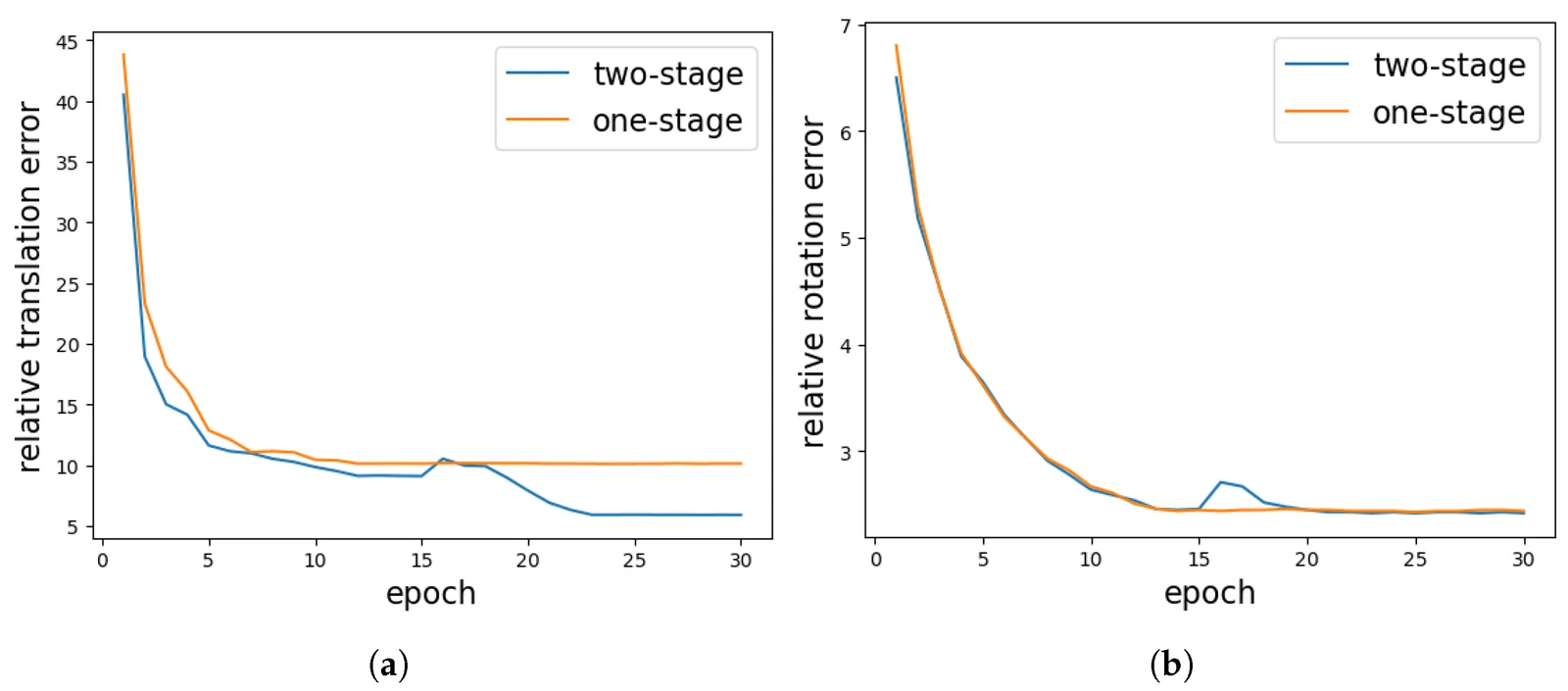
Analysis of the awareness-guided training strategy. (**a**) Relative translation error. (**b**) Relative rotation error.

We compare the proposed two-stage training schedule with one-stage training, in which awareness guidance is applied from the first epoch, as shown in Figure 5. After awareness-guided training is introduced at epoch 15, the two-stage model exhibits a short-term fluctuation, followed by a clear further reduction in translation error, while the one-stage model gradually saturates. The errors become nearly stable after approximately 10 epochs of awareness-guided training. The relative rotation errors of the two strategies converge to similar values. These results support the adopted two-stage training schedule.

Additionally, we summarize the computational complexity and runtime of the main components in Table 1. PoseNet, DepthNet, and FlowNet contain 12.50 M, 12.18 M, and 5.26 M parameters, respectively, with corresponding computational costs of 4.91, 23.41, and 77.63 GFLOPs. Their average runtimes are approximately 6, 43, and 42 ms per frame on an NVIDIA A6000. The observable-static mask is constructed from the predicted pseudo-depth, residual flow, and camera pose after these predictions are detached from the computational graph. Therefore, the mask-construction procedure introduces no additional trainable parameters and does not contribute to gradient backpropagation. It requires only 0.003 GFLOPs and approximately 0.32 ms per frame, indicating that the additional computational cost introduced by the awareness mechanism is relatively small compared with the network components.

### 4.3. Analysis of Geometric and Motion Cues

**Figure 6:**
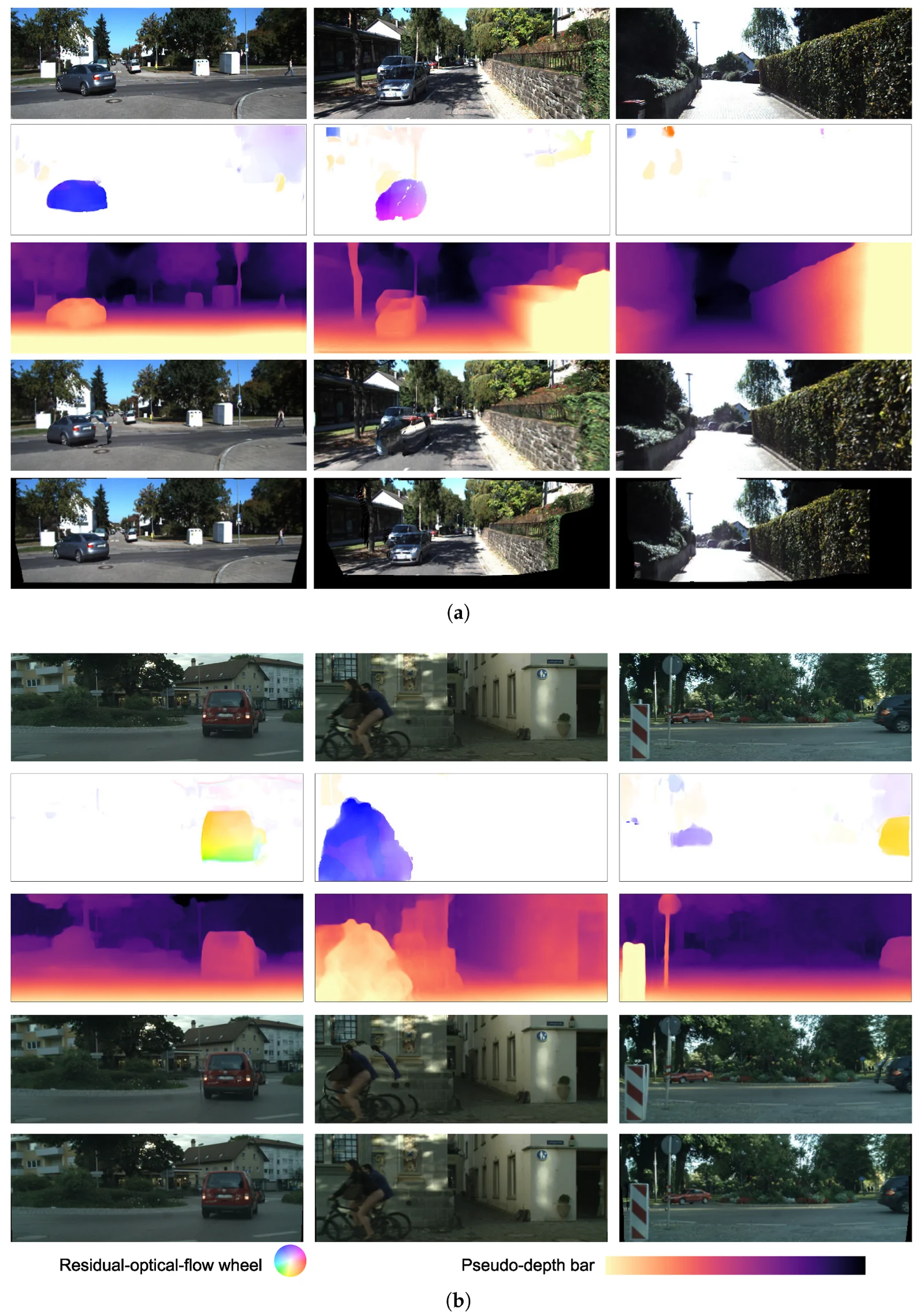
Qualitative results of the learned geometric and motion cues on (**a**) KITTI and (**b**) Cityscapes. From top to bottom, each group shows the input frame, residual flow, pseudo-depth, residual-flow-based reconstructed frame, and pseudo-depth-based reconstructed frame. The KITTI examples are selected from Sequences 08, 09, and 10, while all Cityscapes examples are taken from the validation set.

The learned geometric and motion cues provide the basis for scene awareness and awareness-guided pose estimation. Figure 6 presents qualitative results of the predicted pseudo-depth, residual flow, and reconstructed frames on KITTI and Cityscapes, respectively.

The estimated pseudo-depth provides spatially coherent geometric representations across both KITTI and Cityscapes. Clear relative depth variations can be observed among the road surface, vehicles, vegetation, and distant background regions. In particular, object boundaries and the gradual depth variation along the road are generally well preserved, indicating that the predicted pseudo-depth captures meaningful scene geometry. Such geometric information provides the basis for identifying ground regions and estimating geometrically unreliable or non-observable observations.

The residual-flow maps exhibit localized responses in regions containing independently moving objects. Evident motion responses can be observed around vehicles and cyclists, whereas most static background regions present substantially weaker residual motion. Particularly, the example selected from KITTI Sequence 10 contains no prominent independently moving object, and its residual-flow map correspondingly shows only sparse and weak responses. This contrast indicates that the learned residual flow provides complementary motion information for separating independently moving regions from structures that are largely consistent with the estimated camera motion.

The reconstructed frames preserve most structural details of the original scenes, including road boundaries, vehicles, buildings, vegetation, and roadside objects. The consistency of the reconstructed static regions suggests that the predicted pseudo-depth, residual flow, and camera pose form mutually coherent geometric and motion representations. In contrast, reconstruction quality deteriorates locally around independently moving objects, where noticeable appearance discrepancies and misalignment remain. Directly incorporating these inconsistent reconstruction signals into pose learning may introduce erroneous supervision and interfere with the estimation of rigid camera motion.

Therefore, the proposed self-supervised learning module can extract meaningful geometric and motion cues from unlabeled monocular image sequences. Pseudo-depth provides scene-structure and observability information, while residual flow highlights regions whose motion is inconsistent with the rigid-scene assumption. Their complementary behavior, together with the reduced reconstruction reliability observed in dynamic regions, motivates the subsequent construction of observable static regions and the use of these regions for awareness-guided pose estimation.

### 4.4. Non-Observable Region Detection Based on Pseudo-Depth

To determine an appropriate scaling coefficient for the adaptive depth threshold used in non-observable region detection, we conducted an ablation study with *s* ranging from 1.0 to 1.4. As shown in Table 2, the best performance is obtained at 1.2. A smaller coefficient retains more reprojection-sensitive regions and consequently introduces unreliable information into pose estimation, while an excessively large coefficient may exclude useful geometric observations. Therefore, 1.2 was adopted in all subsequent experiments.

**Figure 7:**
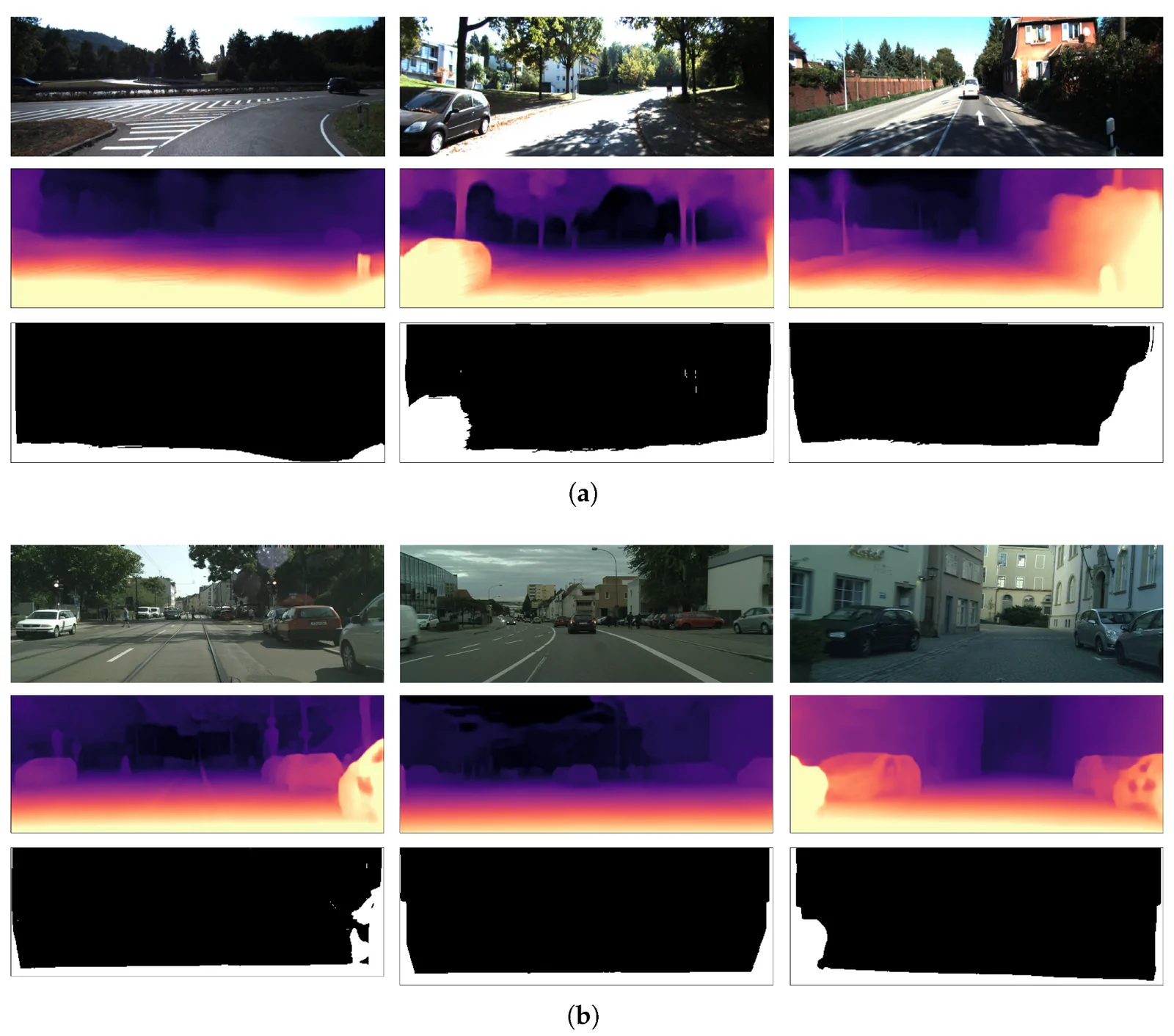
Qualitative results of non-observable region detection on (**a**) KITTI and (**b**) Cityscapes. From top to bottom, each example shows the input image, estimated pseudo-depth map, and non-observable region mask. The KITTI examples are selected from Sequences 01, 02, and 03, while all Cityscapes examples are taken from the validation set.

Figure 7 presents qualitative results of the proposed non-observable region detection. The results on KITTI show that the proposed adaptive threshold adjusts to different road geometries and scene layouts rather than relying on a fixed depth range. For example, nearby road surfaces and objects close to the image boundaries are effectively identified. Similar behavior can also be observed on Cityscapes, although its scenes contain denser traffic, more roadside objects, and more complex urban structures. The detected masks remain consistent with the spatial distribution indicated by the estimated pseudo-depth, demonstrating that the proposed strategy can adapt to different scene configurations.

### 4.5. Dynamic Region Detection Based on Residual Flow

To quantitatively evaluate the effectiveness of the proposed dynamic region detection, we constructed ground-truth motion masks based on the 200 training images of the KITTI semantic segmentation benchmark. The semantic annotations were first used to identify potentially movable object categories, such as vehicles, pedestrians, and cyclists. Since semantic labels alone cannot distinguish moving objects from stationary objects of the same class, the corresponding instances were further inspected across adjacent frames, and only those exhibiting actual motion relative to the surrounding static scene were retained as dynamic regions in the ground-truth motion masks. Stationary instances of the same semantic classes were labeled as static.

We compare the predicted masks with the corresponding ground-truth annotations, as shown in Table 3. For dynamic regions, 99.83% of the pixels are correctly identified, while only 0.17% are misclassified as static. Although 9.09% of static pixels are falsely suppressed, such removal mainly reduces the number of available static observations rather than introducing contradictory motion information. Since pose estimation is constrained by spatially distributed scene structures, the remaining static regions can still provide sufficient information for estimating the global 6 DoF camera motion. Hence, the proposed detector favors effective suppression of dynamic interference, even at the cost of removing a limited portion of static observations.

**Figure 8:**
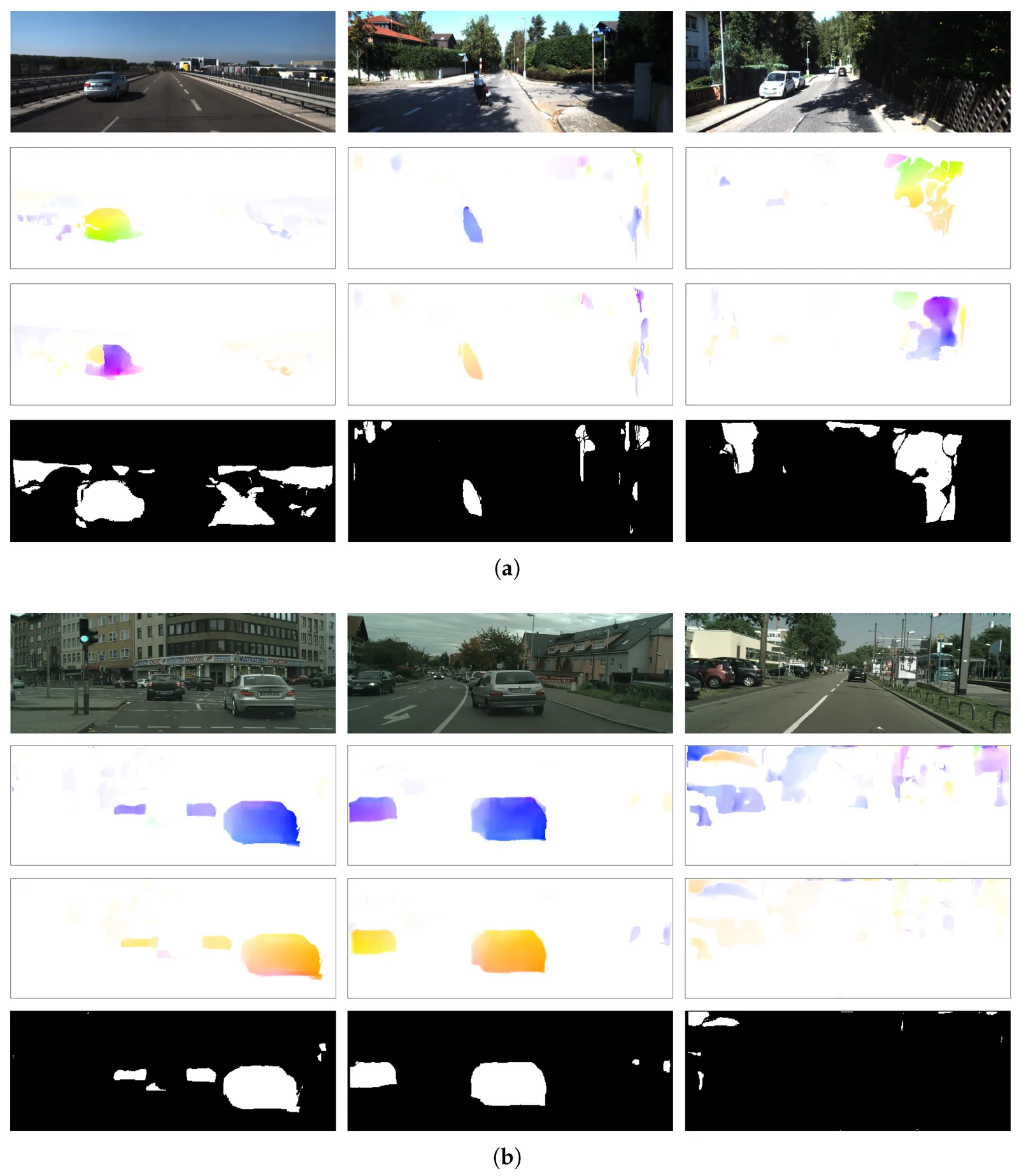
Qualitative results of dynamic region detection on (**a**) KITTI and (**b**) Cityscapes. From top to bottom, each example shows the input image, residual flow $\Delta_{t}^{t-1}$, residual flow $\Delta_{t}^{t+1}$, and dynamic region mask. The KITTI examples are selected from Sequences 01, 02, and 09, while all Cityscapes examples are taken from the validation set.

Figure 8 presents qualitative results of the proposed dynamic region detection. Independently moving objects, such as moving vehicles and motorcycles, are effectively identified as dynamic objects. In contrast, vehicles that are parked are not classified as dynamic simply because they belong to the vehicle category. This behavior indicates that the proposed detector is driven by motion inconsistency rather than semantic cues, thereby preserving stationary vehicles as reliable static observations for pose estimation. However, in the last Cityscapes example, a vehicle traveling in approximately the same direction and at a similar speed as the camera is not detected because its relative motion is too small to produce a distinctive residual-flow response. This remains a limitation of the proposed motion-based detection strategy.

### 4.6. Ablation Study

Table 4 presents the ablation results for the pseudo-depth cue, residual-flow cue, and different awareness injection strategies. Both input-level and loss-level awareness lead to consistent improvements over the baseline, indicating that suppressing unreliable observations is effective at different stages of pose learning. Input-level awareness restricts pose estimation to more reliable visual observations before feature extraction, whereas loss-level awareness reduces the contribution of unreliable regions during optimization. Their joint use further improves the estimation performance, confirming the complementary roles of the two awareness mechanisms.

From the perspective of awareness cues, both pseudo-depth and residual flow independently contribute to improved pose estimation. The pseudo-depth cue identifies regions with unreliable cross-frame geometric observations, while the residual-flow cue captures regions whose motion is inconsistent with the rigid-scene assumption. Combining the two cues produces further improvements under all awareness settings, showing that geometric and motion information provide complementary evidence for identifying unreliable observations.

### 4.7. Ego-Motion Evaluation

We compare the proposed method with several representative approaches, as summarized in Table 5 and Table 6. The results of DualRefine [19] are directly adopted from its original publication. For JPerceiver [18] and Geonet [15], the ego-motion trajectories were obtained using the officially released models, whereas Monodepth2 [14] and the proposed method were trained and evaluated under our experimental setting. To ensure a fair comparison, all reproduced trajectories were aligned using the same procedure employed in DualRefine [19] before computing the corresponding ego-motion metrics.

Table 5 reports the relative translation and rotation errors over KITTI Sequences 00–10. The proposed method achieves competitive performance for both metrics. The improvement is particularly evident in translation, while the improvement in rotation is relatively modest. Thus, the proposed strategy mainly benefits translational motion estimation while maintaining competitive rotational accuracy. The sequence-wise results further show that the proposed method attains the best or second-best performance on most sequences, suggesting that the improvement is consistent across different driving conditions rather than being dominated by a small subset of trajectories.

The absolute trajectory results in Table 6 exhibit a similar trend. The proposed method achieves the lowest average ATE and ARE, with particularly clear gains in ATE. Since absolute trajectory error reflects the accumulated deviation over the entire sequence, the lower ATE indicates that the proposed method is more effective in limiting translational drift during long-term ego-motion estimation. The reduction in ARE is less pronounced, but the overall rotational accuracy remains competitive across the evaluated sequences. These results are consistent with the relative-error evaluation and further confirm that the proposed awareness-guided strategy primarily improves translational estimation and trajectory consistency.

Although the proposed method achieves favorable overall performance, improvements are not uniform across all sequences. In particular, the relative translation and rotation errors on Sequence 05 are slightly higher than those of several competing methods. This regression may reflect the trade-off introduced by the masking strategy: while suppressing unreliable observations reduces misleading supervision, it may also discard some informative geometric constraints for frame-to-frame pose estimation.

**Figure 9:**
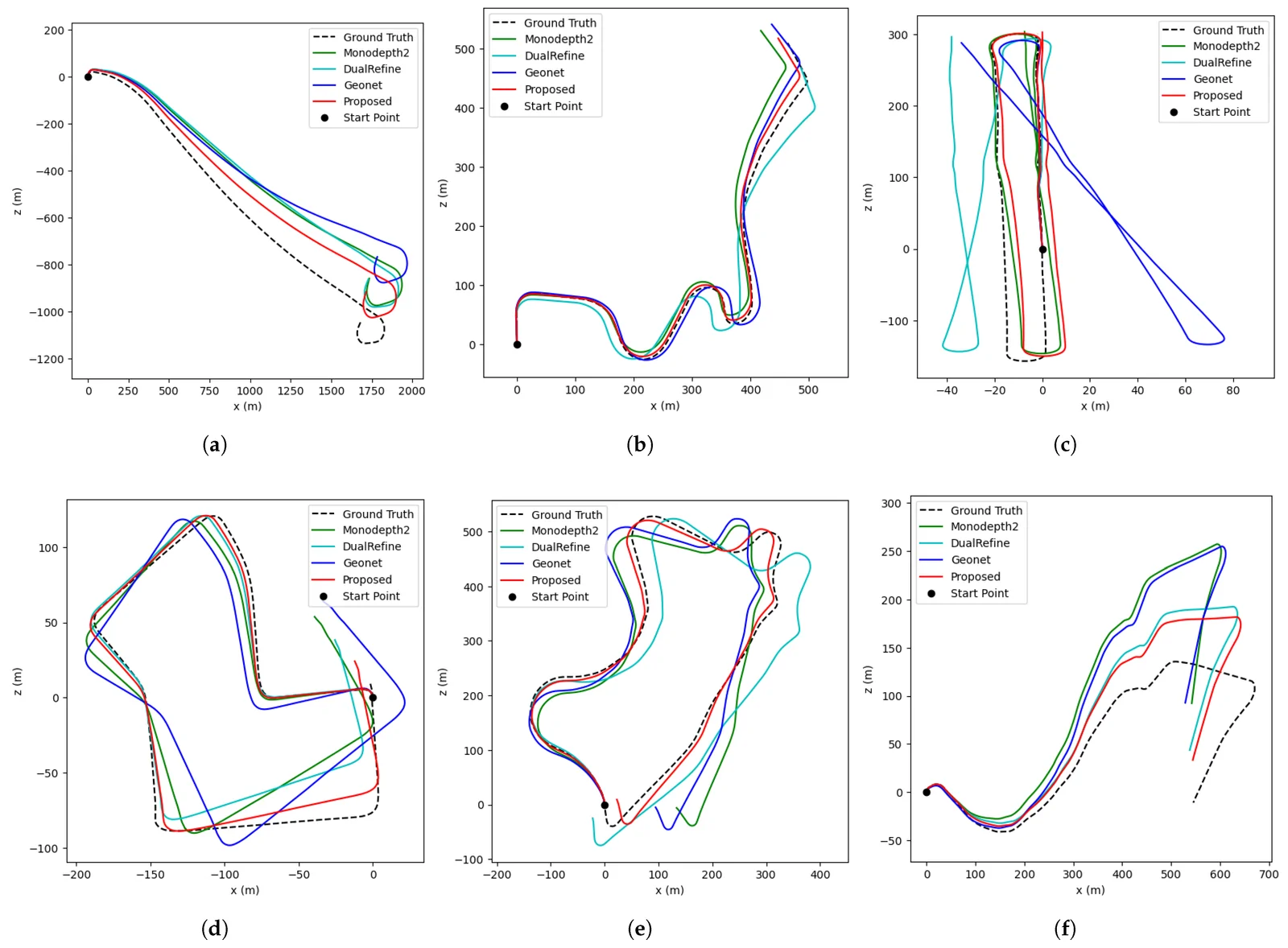
Qualitative results of ego-motion. (**a**) Sequence 01. (**b**) Sequence 02. (**c**) Sequence 06. (**d**) Sequence 07. (**e**) Sequence 09. (**f**) Sequence 10.

Figure 9 presents a qualitative comparison of the estimated trajectories on KITTI sequences. Sequences 01, 02, 06, and 07 are chosen to represent different trajectory geometries, including long-distance motion, curved paths, and repeated turning, while Sequences 09 and 10 are additionally included as the held-out evaluation sequences. To maintain visual clarity and avoid excessive overlap among trajectory curves, the figure presents Monodepth2 [14], DualRefine [19], GeoNet [15], and the proposed method as representative comparisons. JPerceiver [18] is omitted from the trajectory visualization to keep the figure concise, particularly because its quantitative results show a relatively larger deviation from the ground truth in our evaluation.

The proposed trajectories generally follow the ground-truth paths more closely than the competing methods, particularly over long travel distances and in sequences involving substantial changes in heading. This trend is also evident in trajectories containing curved paths and repeated turning motions. Although deviations remain in some challenging segments, the proposed method shows reduced trajectory deformation and endpoint drift in most of the illustrated cases.

## 5. Conclusions

This paper presents an awareness-guided self-supervised monocular visual odometry framework for improving camera motion estimation by suppressing unreliable cross-frame observations. Pseudo-depth is employed to identify non-observable regions, while residual flow is used to capture motion inconsistency caused by independently moving objects. Based on these complementary cues, reliable observable static regions are selected and incorporated into pose learning at both the input and loss levels. In this way, pose estimation is guided by cross-frame observations that are more consistent with the underlying rigid-scene geometry. Qualitative experiments on KITTI and Cityscapes verify the effectiveness of the proposed framework in extracting geometric information from pseudo-depth and dynamic information from residual flow. The visual results show that non-observable regions can be identified according to scene geometry, while independently moving objects can be distinguished through motion inconsistency rather than semantic categories. Quantitative experiments on KITTI further demonstrate that the proposed awareness mechanism improves ego-motion estimation and reduces trajectory errors. The ablation results also confirm that the pseudo-depth and residual-flow cues provide complementary information, and that combining input-level and loss-level awareness leads to further performance gains.

The current framework nevertheless remains limited when independently moving objects exhibit motion close to the camera ego-motion, since their residual motion may become insufficiently distinguishable from the static background. In addition, the use of road-scene geometric assumptions may restrict its applicability to non-road environments. Future work will investigate more robust temporal motion modeling and multi-frame geometric consistency to improve the awareness of reliable cross-frame observations under more challenging motion patterns and diverse scenes.

## Figures and Tables

**Table 1:** Computational complexity and inference time.

Component	Params (M)	FLOPs (G)	Time (ms/Frame)
PoseNet	12.50	4.91	6
DepthNet	12.18	23.41	43
FlowNet	5.26	77.63	42
Mask construction	-	0.003	0.32
Total	29.94	105.953	91.32

**Table 2:** Ablation study on scale coefficient *s* for adaptive depth threshold on Sequences 00–08.

s	1.0	1.1	1.2	1.3	1.4
Avg.$t_{err}$	6.69	6.24	6.18	6.32	6.71
Avg.$r_{err}$	2.55	2.55	2.54	2.54	2.55

**Table 3:** Quantitative evaluation of dynamic region detection.

	Predicted Dynamic	Predicted Static
Ground-Truth Dynamic	99.83%	0.17%
Ground-Truth Static	9.09%	90.91%

**Table 4:** Ablation study of pseudo-depth and residual-flow cues under different awareness strategies for pose estimation. D and OP denote the pseudo-depth and residual-flow cues, respectively. I and L denote input-level and loss-level awareness, while I + L denotes their joint use.

Method	D	OP	AW	Avg.$t_{\mathrm{err}}$	Var.$t_{\mathrm{err}}$	Avg.$r_{\mathrm{err}}$	Var.$r_{\mathrm{err}}$	Avg.ATE	Var.ATE	Avg.ARE	Var.ARE
Baseline				8.94	24.29	2.44	1.76	2.10	1.29	0.31	0.05
Baseline + D	✓		I	8.77	24.05	2.45	1.75	2.09	1.31	0.30	0.06
Baseline + OP		✓	I	8.24	21.89	2.44	1.69	1.97	1.22	0.31	0.06
Proposed	✓	✓	I	8.16	20.53	2.44	1.63	1.89	1.07	0.28	0.05
Baseline + D	✓		L	8.65	23.81	2.44	1.67	1.98	1.25	0.28	0.05
Baseline + OP		✓	L	7.83	19.92	2.42	1.65	1.53	0.69	0.24	0.03
Proposed	✓	✓	L	6.32	18.67	2.42	1.59	1.24	0.46	0.23	0.03
Baseline + D	✓		I + L	7.69	20.73	2.44	1.65	1.64	0.73	0.28	0.04
Baseline + OP		✓	I + L	6.58	13.84	2.43	1.51	1.37	0.57	0.23	0.03
Proposed	✓	✓	I + L	6.04	11.12	2.42	1.25	0.88	0.28	0.20	0.02

**Table 5:** Quantitative comparison of relative translation and rotation errors. Bold and underlined values denote the best and second-best results, respectively.

Seq.	Monodepth2 [14]		JPerceiver [18]		DualRefine [19]		Geonet [15]		Proposed	
	$t_{\mathrm{err}}$	$r_{\mathrm{err}}$	$t_{\mathrm{err}}$	$r_{\mathrm{err}}$	$t_{\mathrm{err}}$	$r_{\mathrm{err}}$	$t_{\mathrm{err}}$	$r_{\mathrm{err}}$	$t_{\mathrm{err}}$	$r_{\mathrm{err}}$
00	15.85	3.03	**3.79**	**1.15**	9.50	3.20	13.72	3.17	8.92	3.83
01	8.26	**0.61**	10.61	2.81	12.05	1.44	8.10	0.81	**3.89**	0.71
02	**4.64**	**1.10**	27.67	3.71	5.87	1.79	6.51	1.49	8.49	1.88
03	10.46	4.55	9.89	4.56	10.55	4.46	10.31	4.46	**3.90**	**2.51**
04	5.24	2.61	4.82	1.79	4.59	2.66	2.65	1.15	**1.27**	**0.57**
05	9.12	3.15	7.31	3.13	7.65	**3.05**	**7.21**	3.08	9.72	3.19
06	2.52	**0.34**	9.16	3.19	7.26	1.91	7.08	1.77	**2.18**	2.64
07	6.34	4.29	10.19	6.86	7.64	4.37	8.86	5.57	**5.08**	**3.88**
08	20.63	2.19	38.10	4.07	13.56	**2.09**	15.19	2.27	**12.15**	3.67
09	8.70	1.77	20.18	4.23	9.06	2.59	**5.85**	**1.32**	6.43	2.13
10	6.54	3.20	15.17	6.61	9.45	4.05	10.21	3.04	**4.44**	**1.61**
Avg.(00–08)	9.23	**2.43**	13.50	3.47	8.74	2.77	8.85	2.64	**6.18**	2.54
Avg.(09–10)	7.62	2.49	17.68	5.42	9.26	3.32	8.03	2.18	**5.44**	**1.87**
Avg.(00–10)	8.94	2.44	14.26	3.83	8.83	2.87	8.70	2.56	**6.04**	**2.42**

**Table 6:** Quantitative comparison of translation and rotation errors. Bold and underlined values denote the best and second-best results, respectively.

Seq.	Monodepth2 [14]		JPerceiver [18]		DualRefine [19]		Geonet [15]		Proposed	
	ATE	ARE	ATE	ARE	ATE	ARE	ATE	ARE	ATE	ARE
00	2.78	0.80	**0.82**	**0.15**	1.93	0.43	2.64	0.83	0.95	0.38
01	4.69	0.08	9.73	0.30	4.68	0.09	6.19	0.13	**2.13**	**0.07**
02	2.59	0.38	8.63	1.47	1.22	**0.28**	2.28	0.39	**0.99**	0.36
03	1.94	0.21	1.57	0.17	1.21	0.22	1.92	0.20	**0.85**	**0.12**
04	1.07	0.09	0.90	0.07	0.51	0.06	0.46	0.03	**0.29**	**0.02**
05	2.28	0.54	1.50	0.38	1.12	**0.33**	1.82	0.43	**0.74**	0.42
06	0.21	**0.01**	0.94	0.13	0.73	0.07	1.15	0.15	**0.13**	0.03
07	0.89	0.25	1.33	0.35	0.58	0.18	1.22	0.36	**0.29**	**0.16**
08	2.40	0.53	3.90	1.06	1.09	**0.23**	2.42	0.51	**1.07**	0.30
09	1.63	0.20	3.49	0.55	**1.15**	0.17	1.36	0.17	1.21	**0.15**
10	2.61	0.30	4.57	0.57	1.43	0.26	2.49	0.25	**1.08**	**0.14**
Avg.(00–08)	2.09	0.32	3.26	0.45	1.45	**0.21**	2.23	0.34	**0.83**	**0.21**
Avg.(09–10)	2.12	0.25	4.03	0.56	1.29	0.22	1.93	0.21	**1.15**	**0.15**
Avg.(00–10)	2.10	0.31	3.40	0.47	1.42	0.21	2.18	0.31	**0.88**	**0.20**

## Data Availability

This study used the publicly available KITTI dataset, which can be accessed at: https://www.cvlibs.net/datasets/kitti/ [50] and https://www.cityscapes-dataset.com/ [51] (accessed on 10 September 2026).
